# Probiotic Characteristics and Antimicrobial Potential of a Native *Bacillus subtilis* Strain Fa17.2 Rescued from Wild *Bromelia* sp. Flowers

**DOI:** 10.3390/microorganisms10050860

**Published:** 2022-04-21

**Authors:** Gabriela N. Tenea, Gabriela Lizeth Gonzalez, Jose Luis Moreno

**Affiliations:** Biofood and Nutraceutics Research and Development Group, Faculty of Engineering in Agricultural and Environmental Sciences, Technical University of the North, Av. 17 de Julio s-21, Barrio El Olivo, Ibarra 100150, Ecuador; glgonzalezc@utn.edu.ec (G.L.G.); jlmoreno@utn.edu.ec (J.L.M.)

**Keywords:** probiotic, *Bacillus subtilis*, antimicrobials, foodborne pathogens

## Abstract

In the present study, we identified the *Bacillus subtilis* strain annotated Fa17.2 isolated from *Bromelia* flower inflorescences collected from the subtropical humid mesothermal region, Santo Domingo de Los Tsachilas Province, Ecuador. The probiotic capacity and antimicrobial potential against four foodborne pathogens were assessed. The cell culture of Fa17.2 is highly resistant to synthetic gastric acid (pH 2.5, 3.0, and 3.5), bile salts (0.3%), tolerating different sodium chloride concentrations (1, 3, and 5%), and growth conditions (15 °C and 45 °C), suggesting its potential probiotic features. The isolate showed no antibiotic resistance and was considered safe as no hemolysis was detected on sheep blood agar. The optimum medium for bacterial growth and the release of antimicrobial compounds was MRS with 10% glucose. The active components released in the neutralized crude extract (NCE) were insensitive to organic solvents, surfactants, and nonproteolytic enzymes and sensitive to proteolytic enzymes suggesting their proteinaceous nature. The antimicrobial activity was enhanced by heat and maintained active over a wide range of pH (2.0–8.0). Moreover, the crude extract (CE) showed inhibitory activity against several Gram-negative and Gram-positive bacteria. The molecular weight of partially purified precipitated bacteriocin-like substances (BLISs) was about 14 kDa in 20% Tricine-SDS-PAGE. The CE obtained from Fa17.2 inhibits the growth of four foodborne pathogens, *Staphylococcus aureus*, *Escherichia coli*, *Kosaconia cowanii*, and *Shigella dysenteriae*, which implies its potential as an antimicrobial producer strain.

## 1. Introduction

Worldwide, millions of individuals suffer from gastrointestinal problems, most of them due to the consumption of contaminated food and water [1]. Ecuador makes no exception. Throughout 2019, foodborne pathologies reached 19,487 cases, while in 2021 a decrease in about 60% was reported [2]; however, there are still cases due to lack of knowledge of health standards, and handling and conservation of food by sellers, to guarantee the quality of the products that are marketed. This decrease may be related to the pandemic disease of the coronavirus, as street sales were blocked. Nonetheless, in most cities, the lack of an appropriate structure in the retail markets might be the main cause of the contamination; however, the products failed to reach the required quality [3]. In addition, the use of beneficial microorganisms such as probiotics in foods that contain antimicrobial substances is very limited in the Ecuadorian market. Probiotics are defined as live microorganisms that are administered to hosts in adequate amounts to improve human health [4]. However, only a few products contain such microorganisms as *Lactobacillus rhamnosus* GG (ATCC53100), a commercial strain [5].

Customer demand for high-quality, free of chemicals or antibiotics containing foods is increasing; therefore, the identification of natural alternatives using beneficial microorganisms, or their derivatives, can be a solution. In the last decade, several researchers have investigated the use of antimicrobials fabricated by food-grade microorganisms, such as peptides or proteins with antimicrobial activity that is secreted into the extracellular matrix during the metabolic process of various bacteria, which can prevent the increase in single or combined pathogens; they are easily degraded by enzymes, thus do not affect the human body pathogenically [6]. Although many bacterial species generate antimicrobials, only a few are applied to foods as biological preservatives [7]. 

One of the best-studied Gram-positive bacteria, *Bacillus subtilis*, an aerobic or facultatively anaerobic bacterium, is considered a model of cell differentiation and industrial exploitation [8]. Currently, various commercial formulations contain bacilli as active ingredients, thanks to their ability to colonize, reproduce easily, and their high stability concerning endosporogenesis; this last characteristic is especially essential, as it allows them to survive in stressful situations, such as abiotic conditions, that facilitate long term production and storage [9]. These species remain widely distributed in a wide diversity of habitats, integrated freshwater, rhizosphere, marine, and terrestrial ecosystems, and their species are commonly associated with plants [10]. *Bacillus subtilis* are recognized as safe and reliable probiotic strains that are non-pathogenic to humans and animals [11]. Antimicrobial metabolites are generated during their growth and reproduction [12].

Several registered strains have been marketed as probiotic supplements for human consumption in Asia, Europe, and the US [13]. Feed supplementation with spores can provide numerous benefits including animal improvement in digestibility and immune modulation [14]. The spores are metabolically quiescent and should be in a metabolically active state to perform certain probiotic functions such as secretion of antimicrobial compounds and enzymes, and synthesis of short-chain fatty acids [15,16]. *B. subtilis* gained more interest to be used as a probiotic, and their consumption in foods is believed to be associated with numerous health benefits, such as increased immunity, reduced bone loss in postmenopausal women, and antiallergic effects [17]. In addition, *Bacillus* isolates are well-known for producing a wide range of antimicrobial compounds, including lipopeptides and BLIS [18]. The main types of antimicrobial compounds from *B. subtilis* comprise peptides such as lantibiotics and lantibiotic-like peptides, and non-peptide compounds such as polyketides, an amino sugar, and phospholipid [19]. 

The increase in the value of biological diversity and the exceptional richness of tropical forests improve the chances of the systematic use of genetic resources or their derivatives in various biotechnological processes. Ecuador has not yet used its genetic resources effectively. There are no studies that quantify values in the bioproducts market. In the case of biological products, research and development activities are concentrated in multinational companies. From free of board (FOB), the annual import values of these products are close to USD 250 million; therefore, there is a huge opportunity to enter this market with innovative technologies, such as probiotics, nutraceuticals, and derivatives developed by the researchers and transferred to different productive sectors of the Andean region [20]. Given the extensive changes made by Ecuadorian government policies, some natural areas such as subtropical forests have been considered relevant genetic resources for biotechnological research. However, to detect and characterize new bacterial species, the bacterial microbiota associated with flowers and fruits was studied [21]. Particularly, lactic acid bacteria (LAB) associated with these micro-niches were investigated [21]. We taught that the strains associated with these extreme niches may allow selecting more robust strains with broad antimicrobial capacity against foodborne pathogens as well as native strains with probiotic potential. Throughout the selection process for isolates showing antimicrobial capacity against at least two Gram-negative bacteria (*Salmonella enterica* and *E. coli*), one bacillus showing sporulation “escapes” along with other isolated lactobacilli from the selection on MRS media. Moreover, these isolates during cultivation were characterized by a “particular flower-fragrance” which might be linked with the secretion of some volatile compounds that need further attention. At this point, we speculate that this feature might be connected to the *Bromelia* flower origin. Due to its comparable inhibitory activity with lactobacilli, we selected this isolate for further taxonomic identification and evaluated its probiotic capacity and antimicrobial potential against some foodborne pathogenic bacteria. Therefore, we performed various in vitro studies to test their tolerance to intestinal gastric acid, bile salts, and sodium chloride, as well as different growth temperatures, and hemolysis and antibiotic susceptibility for safety issues. In addition, the effect of medium composition on the production of antimicrobial substances as well as the antimicrobial spectrum was evaluated against several Gram-positive and Gram-negative bacteria. Moreover, the nature of these antimicrobials was evaluated in vitro along with their sensitivity to various pH, heat, inorganic, and organic treatments. Moreover, the partially precipitated BLIS were analyzed by Tricine-SDS-PAGE to estimate their molecular weight. 

## 2. Materials and Methods

### 2.1. Sampling, Bacterial Isolation, and Identification 

Samples consisting of flower inflorescences of *Bromelia* sp. were collected aseptically from a subtropical humid mesothermal region of Santo Domingo de Los Tsachilas Province, 43 km away from Quito, the capital city. Samples were packaged in clean bags, then stored at 4 °C for further analysis. The isolation and selection procedures were performed as described earlier [21]. One isolate assigned Fa17.2, showing spore formation, was selected based on its capacity to inhibit *Salmonella enterica* subsp. *enterica* ATCC 51741 and *E. coli* ATCC25922. The BBL Crystal Gram-positive identification system (cat # 245010, BD Company, Franklin Lakes, NJ, USA), a miniaturized identification method using 29 enzymatic and biochemical substrates, was used for genera classification according to the manufacturer’s instructions. Moreover, 16S rRNA gene sequencing was used for taxonomical classification following a standard procedure (Macrogen Inc., Seoul, Korea). The microorganism culture was preserved by deep freezing in glycerol solution before use in further analyses. 

### 2.2. In Vitro Probiotic Feature Assessment

#### 2.2.1. Survival under Gastric Juice Conditions

Survival was determined using 8 log CFU/mL of the overnight culture of Fa17.2 by the plate-agar method using the MRS agar medium (MRS, Difco, Detroit, MI, USA). Briefly, after incubation at 37 °C (with shaking 200 rpm), the bacterial cells were harvested at 5000× *g* for 5 min at 4 °C, the biomass was rinsed twice with sterile Ringer’s solution (pH 7.2) and resuspended in synthetic gastric juice solution with the established pH of 2.5, 3.0, and 3.5 followed by incubation for 4 h at 37 °C. The cell viability was determined at intervals of 1 h by counting the cells on the MRS agar. The gastric juice was formulated as follows: glucose (3.5 g/L), NaCl (2.05 g/L), KH_2_PO_4_ (0.60 g/L), CaCl_2_ (0.11 g/L), and KCl (0.37 g/L), adjusted to corresponding pH using 1 M HCl. After autoclavation at 121 °C for 15 min, porcine bile (0.05 g/L), lysozyme (0.1 g/L), and pepsin (13.3 mg/L) were added as stock solutions before analysis [22]. Components were obtained from Sigma Aldrich (Sigma Aldrich, St. Louis, MO, USA). The % of cell survival was calculated as follows: ((cell counts at initial incubation time − cell counts at the final incubation time)/cell counts at the initial time) × 100). The results were compared with a probiotic reference strain, *Lactobacillus acidophilus* ATCC4846 (LA). 

#### 2.2.2. Survival under Bile Conditions 

In the case of bile, the overnight Fa17.2 cell culture (8 log CFU/mL) was incubated in MRS broth containing 0.3% bile salt (oxgall, *w*/*v*) at 37 °C for 4 h (with shaking, 200 rpm). The cell viability was determined by plating 100 μL bacterial cells on MRS agar (MRS, Difco, Detroit, MI, USA). The % of cell survival was calculated as indicated in Section 2.2.1. No modified MRS broth was used as control and the experiment was run in triplicates starting from different batches of culture. The results were compared with the probiotic LA reference strain. 

#### 2.2.3. Optimum Temperature and Growth Tolerance in the Presence of Sodium Chloride

Overnight culture (8 log CFU/mL) of Fa17.2 was inoculated in tubes containing MRS broth and incubated at 15 °C and 45 °C for 24 h (with shaking, 200 rpm), and the absorbance at 605 nm was measured at the initial and final incubation time. Similarly, the tolerance in the presence of sodium chloride was evaluated upon the inoculation of the overnight culture of Fa17.2 in broth medium containing 1%, 3%, and 5% sodium chloride (*w*/*v*) for 24 h at 37 °C. Cell growth was monitored for each treatment and the effect of sodium chloride on cell survival was determined using the plate-agar method. The % of cell survival was determined and is described in Section 2.2.1. No modified MRS broth was used as control and the experiment was run in triplicate starting from individual batches of bacterial culture. The results were compared with the probiotic LA reference strain. 

#### 2.2.4. Hemolysis Test 

The hemolytic activity of the isolate was determined as previously described [23]. The Columbia agar containing 5% (*w*/*v*) sheep blood was used. After incubation at 37 °C for 48 h, the hemolytic activity was evaluated and classified based on the lysis of red blood cells in the medium around the colonies: the green zones around colonies (α-hemolysis), clear zones around colonies (β-hemolysis), and no zone around colonies (γ-hemolysis). The strain is considered safe if γ-hemolysis was detected. 

#### 2.2.5. Antibiotic Susceptibility

Susceptibility to several antibiotics was determined using commercial disks of ampicillin, gentamicin, kanamycin, amoxicillin/clavulanic acid, tetracycline, and cefuroxime at the concentrations recommended by the Scientific Committee on Animal Nutrition (disks provided by Merck, Darmstadt, Germany) by the disk diffusion assay as described in [21]. The experiment was run in triplicate starting with different batches of bacteria culture and the disks were verified by *E. coli* ATCC25922, a reference strain for quality control. Using a similar approach, the minimum inhibitory concentration (MIC) distribution within the bacillus group was measured using the E-test (Biomerieux, Durham, NC, USA, E-test) assay following the manufacturer’s instructions. The microbiological breakpoints reported by the FEEDAP document were used to categorize bacilli as susceptible or resistant. The strains showing a MIC higher than the EFSA breakpoint were considered resistant [24]. 

### 2.3. Characterization of Antimicrobial Substances Produced by Fa17.2 In Vitro

#### 2.3.1. Preparation of CE and Antimicrobial Assay 

CE from the target strain was obtained as described in [25]. In brief, the CE was recovered by centrifugation (13,000× *g* for 20 min at 4 °C) and filtration using a 0.22 μm porosity syringe filter (# STF020025H, ChemLab Group, Fort Smith, AR, USA) of an overnight culture of Fa17.2. The indicator strain (100 μL) grown in broth medium (7 log CFU/mL) was mixed with 3.5 mL of soft MRS agar (0.75%), overlaid on nutrient agar plates, and incubated at 37 °C for 2 h. The CE (100 μL) were transferred onto wells (6 mm) on overlaid agar, incubated at 37 °C, and subsequently examined for inhibition zones at 48 h. The experiments were run in triplicate and the mean value of the inhibition zone was determined. As indicator microorganisms: *S. aureus* ATCC1026, *S. dysenteriae* UTNFa37-1, *K. cowanii* B2Sh1 (laboratory isolate), and *E. coli* ATCC25922 were used. Each experiment was performed in triplicates starting from individual bacterial cultures. 

#### 2.3.2. Effect of Medium Composition on Antimicrobials Production 

The effect of medium composition on bacterial growth and the release of antimicrobial compounds was evaluated. The following media were tested: (1) MRSS: MRS broth supplemented with sucrose (5, 10, 20, 40, and 50%); (2) MRSG: MRS broth supplemented with glucose ((5, 10, 20, 40, and 50%); (3) and MRSGly: MRS broth supplemented with glycerol (5, 10, 20, 40, and 50%). As a control, MRS broth without additional nutrients was used. The Fa17.2 strain was inoculated individually in each medium combination for 24 h and the CE obtained as mentioned above was used in the agar-well diffusion assay. As an indicator, the microorganisms described in Section 2.3.1 were used. Each experiment was performed in triplicates starting from individual bacterial cultures. 

#### 2.3.3. Evaluation of Antimicrobial Spectrum of Inhibition

The antimicrobial activity of the CE was obtained after growing the bacteria in the optimum medium detected for each target bacteria. The results were compared with the antimicrobial activity obtained from bacteria grown in MRS media (−) with no additional nutrients. The percentage of antimicrobial activity change was calculated as follows: ((average diameter of inhibition zone of CE obtained from the optimum media—average diameter of inhibition zone of CE obtained from MRS (−)/average diameter of inhibition zone of CE obtained from MRS media (−)). The bacteriocinogenic strain *L. plantarum* ATCC8014 (LP) was used as a reference. The indicator strains of Gram-positive: *S. aureus* ATCC1026, *Lactococcus lactis* ATCC11474, *L. acidophilus* ATCC4358, *Bifidobacterium brevis* ATCC15700, and *Streptococcus thermophilus* ATCC19298; and Gram-negative: *Shigella* ssp. UTNShg1 (laboratory strain), *S. enterica* subsp. *enterica* ATCC51741, *Salmonella* ssp. UTNSm2 (laboratory strain), *S. sonnei* ATCC25931, *S.* *dysenteriae* UTNFa37-1 (laboratory strain), *E. coli* ATCC25922, *E. coli* ssp. UTNEc2 (laboratory strain), and *K. cowanii* B2Sh1 (laboratory strain) were used. 

#### 2.3.4. Estimation of the Chemical Nature of CE

The CE were submitted to different treatments as described previously [25]. Briefly, aliquots of CE were treated 10 min at 80 °C and pH 6.0 to rule out the effect of acids on the antimicrobial activity (NCE). Moreover, NCE was treated with catalase enzyme (1 mg/mL) to prevent the possible inhibitory of hydrogen peroxidase. Furthermore, NCE was independently treated with proteinase K, pepsin, lysozyme, and α-chymotrypsin (Sigma-Aldrich Corporation, St. Louis, MO, USA) at the final concentration of 1 mg/mL, incubated for 2 h at 37 °C and 5 min at 100 °C for enzyme inactivation. All experiments were run in triplicate using *S. aureus* ATCC1026, *S.* *dysenteriae* UTNFa37-1, *K. cowanii* B2Sh1, and *E. coli* ATCC25922 as indicator strains. The control for all experiments was the sterile MRS medium. 

#### 2.3.5. Effect of Heat, pH, and Detergents on Antimicrobial Activity

Aliquots of CE were incubated for 10, 30, and 60 min at 60, 80, 90, and 100 °C as well as 15 min at 121 °C (autoclaving). In another batch, aliquots of CE were adjusted at pH 2.0, 4.0, 6.0, and 8.0, incubated for 3 h at room temperature. In addition, the effect of Triton X-100 (BDH Chemicals Ltd., Poole, UK), sodium dodecyl sulphate ((SDS) Sigma-Aldrich Corporation, St. Louis, MO, USA), and ethylenediaminetetraacetic acid ((EDTA) Sigma-Aldrich Corporation, St. Louis, MO, USA) at the final concentration of 1 mg/mL was evaluated. All experiments were run in triplicate using *S. aureus* ATCC1026, *S.* *dysenteriae* UTNFa37-1, *K. cowanii* B2Sh1, and *E. coli* ATCC25922 as indicator strains. The control for all experiments was the sterile MRS medium. 

### 2.4. Bacteriocin Molecular Size Approximation

To obtain the precipitated bacteriocin, the CE was treated with 80% ammonium sulfate, incubated overnight at 4 °C, and centrifuged at 10,000× *g* for 30 min. The bacteriocin was recovered in ammonium acetate 25 mM (pH 6.5) and desalted using a midi dialysis kit (cat # PURD10005-1KT, Sigma-Aldrich, St. Louis, MO, USA) pre-equilibrated with phosphate buffer (pH 7.0) and stored at −80 °C before use. The bacteriocin molecular weight was determined by the Tricine-SDS-PAGE method using pre-casted acrylamide gels (4–20%) and a mini-vertical electrophoresis system (Expedeon, Abcam, Cambridge, MA, USA). The broad range protein molecular marker (cat # V8491, Promega, Madison, WI, USA) was used for molecular weight determination. The gel was stained with InstantBlue ready-to-use stain (Expedeon, Abcam, Cambridge, MA, USA) for 2 h and distained with a solution of 30% methanol (*v*/*v*) and glacial acetic acid, 10% (*v*/*v*) until the bands became clear.

### 2.5. Statistical Analysis

The means were calculated from repeated measurements performed three times. For the antimicrobial activity, the effect of the medium and enzymes, one-way analysis of variance (ANOVA), and Tukey’s post hoc test were used to determine significant differences between the means. For the effect of heat, and detergents, the ANOVA with a split-split-plot experimental design was performed. Then, Duncan’s multiples tests and Least Significant Difference with Bonferroni correction (LSD) were applied to determine significant differences between the means. The statistical significance used was *p* < 0.05 (SPSS version 10.0.6, IBM, Armonk, NY, USA).

## 3. Results and Discussion

**Figure 1:**
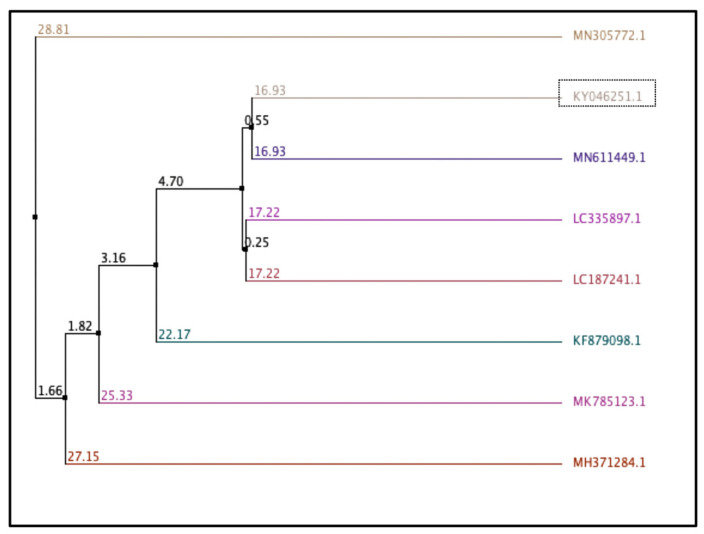
Average distance calculated based on percentage of similarity between sequences. Bacillus strains from the database and the contig 16S rRNA sequence of Fa17.2. Trees were calculated based on a measure of similarity between each pair of sequences in the alignment: PID. The percentage identity between the two sequences at each aligned position. The number on the branch is the bootstrap value that indicates the extent of relatedness between two subjects. Legend: KY046251.1: *B. subtilis* strain Fa17.2; MH371284.1: *B. subtilis* strain C1; MK785123.1: *B. vallismortis* strain VS-5; LC335897.1: *B. subtilis* PH; KF879098.1: *Bacillus* spp. BAB-2797; MN305772.1: *B. subtilis* strain OTG009; MN611449.1: *B. subtilis* strain *subtilis*; LC187241.1: *Lactobacillus murinus* strain LAP1.

Based on the 16S rRNA gene sequences, a comparative sequence analysis, and a biochemical characteristics analysis, the new isolate assigned Fa17.2 belongs to the genus *Bacillus* with 99% identity to *Bacillus subtilis*. The strain was registered at GenBank with the accession number KY046251.1 (1 November 2016). Using multiple sequences alignment with Jalview (version 2.11.2.0) [26], the average distance was calculated from the percentage of identity between the sequences of some *Bacillus* strains retrieved from the database and the contig sequence of the target strain (Fa17.2). (Figure 1). The closest genome to Fa17.2 was *B. subtilis* strain *subtilis* (MN611449.1). 

### 3.1. Assessment of Probiotic Characteristics of B. subtilis Strain Fa17.2

#### 3.1.1. Tolerance to Gastric Juice and Bile Salts

**Figure 2:**
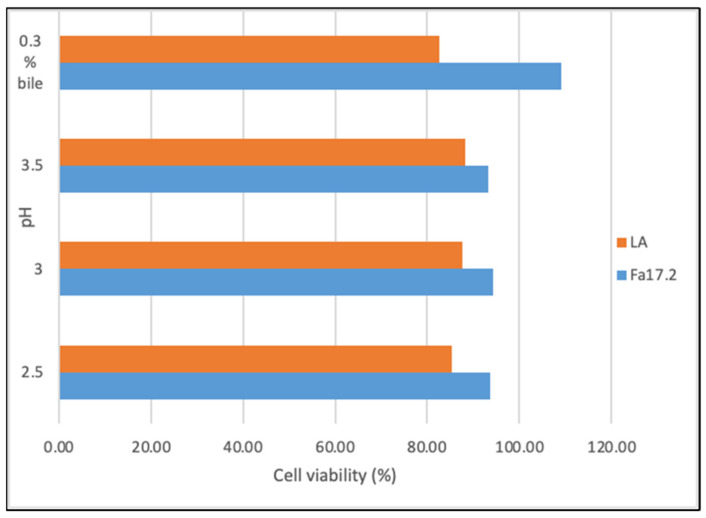
Cell viability (%) upon incubation with gastric juice at different pHs and bile salt (0.3%). Legend: Fa17.2: *B. subtilis* Fa17.2; LA: *L. acidophilus* ATCC4846.

To exert their probiotic potential, the new bacterial isolates should present resistance to gastric acid and bile [27]. Figure 2 shows the cell viability (%) of both strains after 4 h of incubation with gastric juice at pH 2.5, 3.0, and 3.5. The initially inoculated population at the time 0 (hours) and during 4 h of incubation is shown in Table S1. At pH2.5, the registered percentage of decrease was 6.31% for *B. subtilis* Fa17.2 and 14.66% for the reference probiotic strain *L. acidophilus* ATCC4846. Similarly, cell viability decreases of 5.71% and 12.43% were registered at pH 3.0, and 6.79% and 11.75% at pH 3.5 for Fa17.2 and LA, respectively, upon 4 h of incubation. Although a percentage decrease in the growth of *Bacillus* spp. was evidenced at pH 3.0, by increasing the pH (4.0), an increase in cell viability was observed. A similar study indicates that the vegetative cells of *B. subtilis* DET6 and *B. megaterium* JHT3 had poor resistance to artificial gastric acid, whereas the spore cells were resistant to gastric conditions [28]. In addition, the resistance to synthetic gastric acid of two *B. subtilis* CBD2 and KMKW4 strains isolated from Korean fermented foods were demonstrated [10]. At 4 h, a significant increase (*p* > 0.05) in the cell population of Fa17.2 was observed when incubated with 0.3% bile salts (oxgall, *w*/*v*), while the cell population of the reference LA strain decreases (Table S1). In a previous analysis, several selected LAB strains isolated from wild fruits and flowers showed high bile resistance with a significant increase in the cell population at 4 h of incubation, thus suggesting that bile might stimulate cell growth [21]. In another study, an increase in *B. subtilis* P223 vegetative cells in bile salts (0.3%) was registered [29]. Based on these results, we suggest that *B. subtilis* Fa17.2 can resist acidic gastric conditions; therefore, it might pass through the intestinal gut, an important criterion when selecting potential probiotic strains. Moreover, the bile stress did not affect the growth of Fa17.2 cells, while the reference commercial probiotic cells were less tolerant. We suggested that this property is species-specific and might be connected to the origin (tropical flower), but this statement needs further investigation. 

#### 3.1.2. Tolerance to Sodium Chloride and High-Temperature Growth Conditions

**Figure 3:**
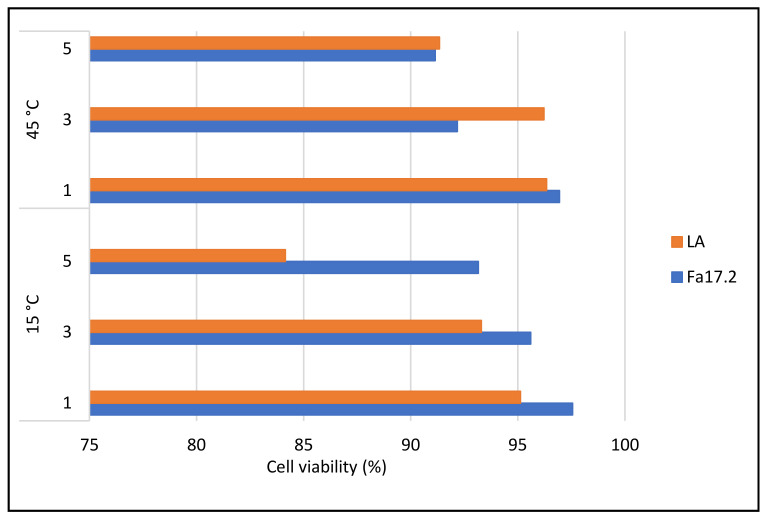
Cell viability (%) in different concentrations of NaCl upon 24 h of incubation at 15 °C and 45 °C. Legend: Fa17.2: *B. subtilis* Fa17.2; LA: *L. acidophilus* ATCC4846.

Temperature is one of the most important factors that affect the growth and survival of microorganisms, it varies between different genera and reflects the optimal temperature range of its natural habitat [28]. In this study, the results indicated that both strains grow at 15 °C and 45 °C and tolerate different concentrations of sodium chloride (Table S2). At 15 °C a lower decrease in cell population was observed for the native Fa17.2 strain compared with reference LA, while at 45 °C, the strains showed a comparable tolerance profile (Figure 3). In a similar study, less tolerance at high temperature (43 °C) and salt concentrations (2, 4, 7, and 10%) was noticed for different *Bacillus* strains [29]. Our results agreed with previous studies showing that sodium chloride tolerance might be strain-dependent [21,29]. 

#### 3.1.3. Antibiotic Susceptibility and Pathogenicity

Antibiotic susceptibility of bacterial strains intended for use as probiotics must be confirmed for safety proof [30]. Antibiotic tolerance can help balance the gut microflora after antibiotic administration [31]. The main concern about using *Bacillus* spp. as probiotics is due to their ability to transfer antibiotic resistant genes [32]. In addition, *Bacillus* spp. do not belong to the commensal microbiota of the digestive tract; however, several strains of the genus are integrated into food supplies [33]. Moreover, some strains of *Bacillus* are used as feed additives, biomass for animal feed consumption, or enzyme/vitamin production [34], and many species have been added to the EFSA QPS list [35]. Based on disk diffusion agar assay results, the strain Fa17.2 was sensible to all antibiotics except gentamycin (data not shown). According to EFSA [24], *Bacillus* strains are listed as resistant to all antibiotics except ampicillin. The selected Fa17.2 does not show ampicillin resistance. In a similar study, *B. clausii* ATCC700160 and *B. subtilis* P223 strains were found resistant to streptomycin [29]. The microbiological breakpoints reported by the FEEDAP were used to categorize bacilli as susceptible or resistant [24]. In this study, the E-test assay confirmed that the strain was sensible to the antibiotics tested. In addition, the resistance showed by Fa17.2 to gentamycin was not confirmed by the E-test analysis (Table S3). Moreover, the strain did not show any hemolysis on sheep blood agar, indicating that the strain is not pathogenic. 

### 3.2. Assessment of Inhibitory Capacity and Characterization of Antimicrobial Substances Produced by Fa17.2

#### 3.2.1. Culture Medium Optimization to Enhance the Bacteria Growth and Antimicrobials Production 

The agar diffusion analysis was carried out to identify the effect of cell growth in several culture media on the inhibitory activity against four indicator strains. The average mean values registered against all indicator strains are presented in Tables S4–S7. The results indicated that all media tested displayed favorable inhibitory effects against all indicator strains. Nonetheless, the greater inhibitory effect of CE Fa17.2 against *S. aureus* ATCC1026 was detected after growing in MRS media supplemented with 10% and 20% glucose (Table S4). Comparable results were obtained against *E. coli* ATCC25922 when growing Fa17.2 in MRS supplemented with 10% glucose (Table S5). Moreover, the results indicated that against the B2Sh1 strain the optimum media for Fa17.2 to exert maximum inhibitory activity were MRS supplemented with 20% glycerol and 10% glucose (Table S6). The MRS media supplemented with 10% glucose had a favorable effect on the production of antimicrobial compounds for the Fa17.2 strain against *S. dysenteriae* Fa37-1 (Table S7). Likewise, the reference strain LP showed greater activity in medium supplemented with 20% glycerol against all tested indicator strains. Supplementation of MRS with different concentrations of carbohydrates provided an increase in yield which varies depending on the type of sugar used. Although several media compositions showed differences in the growth of the cells and coupled with antimicrobial activity against the four pathogens, the optimum media chosen for the target strain were MRS with 10%. Sucrose also served as a carbon source for optimal growth; however, did not show the same antimicrobial effect against all indicator bacteria, while glycerol appears to be deficient in growth. The optimum media for the bacteriocin production of LP were MRS with 20% glycerol. Early research showed the positive effect of a medium supplemented with different sugars (glucose, sucrose, and xylose) on the *B. subtilis* growth, and an enhanced antimicrobial activity was correlated with the accumulation of cell biomass [36]. In another study, Monteiro et al. [37] found that for *B. subtilis*, glucose exerted an inhibitory effect on spore production if its concentration exceeded 20 g/L. However, the results agreed with other studies indicating that the inhibitory efficiency depends on the medium composition and pathogen. 

#### 3.2.2. Inhibitory Spectrum 

**Figure 4:**
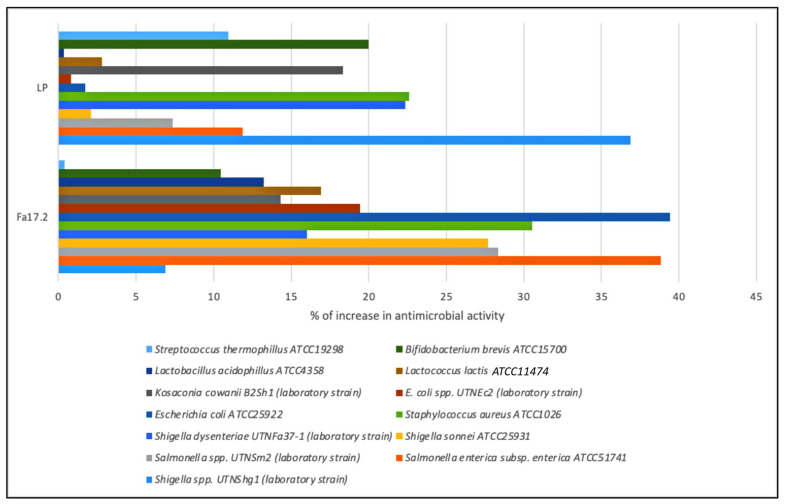
Percentage of increase in the inhibitory activity of CE obtained from Fa17.2 and LP after growth in optimum media versus control (MRS control).

In this study, the inhibitory activity of CE obtained from Fa17.2 and the reference LP strains was evaluated against several indicator strains. The percentage of inhibitory activity changes (%) is shown in Figure 4. Although both strains showed inhibitory activity against all tested indicator strains, the highest activity was registered by the LP strain against *Shigella* UTNShg1. Within the Fa17.2 group, the most sensitive strains were *S. enterica* ATCC51741, *S. aureus* ATCC1026, *E. coli* ATCC25922, and *Salmonella* UTNSm2 (*p* > 0.05). Lower activity was detected against *Lactobacillus* and *Bifidobacterium* strains. An early study indicated a higher inhibitory capacity of a cell-free supernatant extracted from the *B. subtilis* KKU213 strain against several Gram-positive pathogens such as *B. cereus*, *S. aureus*, and *L. monocytogenes*, with the low levels of activity against lactic acid bacteria, *E. faecalis* BT2, and MG30 [38]. In another study, antibacterial substances produced by *B*. *subtilis* LFB112 isolated from Chinese herbs were effective against Gram-positive and Gram-negative bacteria involved in domestic animal diseases, including *E. coli*, *S. pullarum*, *Pseudomonas* ssp., *Clostridium perfringens*, *Micrococcus luteus*, *S. bovis,* and *S. aureus* [38]. Due to its nature as an endospore former strain, the ability of *B. subtilis* to resist harsh environments and produce cocktails of antimicrobial substances such as bacteriocins and lipopeptides is an advantage to stimulate the inhibition of pathogenic bacteria. In the industry, the control of these microorganisms is of vital importance, so these strains represent a great option to be used as natural biopreservatives [39]. Moreover, endospore formation allows it to withstand extreme stresses and offers biological solutions to formulation conservation problems when produced on an industrial scale [40]. Genome sequencing highlighted the genus *Bacillus* as an unexpected source of antimicrobial compounds including surfactin, fengycin, iturin, mycosubtilins, and bacillomycins, which are amphiphilic, membrane-active biosurfactants [41]. These molecules might contribute to the overall inhibitory action. Further investigations are required to identify the antimicrobial molecules produced by the target Fa17.2 as well as their mode of action against pathogenic strains. 

#### 3.2.3. Detection of the Nature of Antimicrobial Substances

Table 1 shows a comparison of the averages of the diameter of the inhibition zone obtained at 48 h of incubation for different treatments of NCE with proteolytic and non-proteolytic enzymes against the indicator bacteria under study. However, the treatment with proteinase K (1 mg/mL) resulted in completely abolishing the antimicrobial activity, suggesting the protein-like nature of compounds released in the CE. The treatment with alpha-chymotrypsin, pepsin, and trypsin resulted in a little decrease in inhibitory activity (*p* < 0.05), while the treatment with lysozyme did not show any change in the antimicrobial activity. Thus, the substances released in the CE might not be affected by the presence of these enzymes in the medium. The treatment with catalase resulted in the reduction in the inhibitory effect indicating that the activity might be hydrogen peroxide dependent. Similar results were observed in the case of *B. subtilis* strain RLID 12.1 isolated from the soil when a decrease in activity was shown after treatment with proteinase K (10 mg/mL), while the activity was partially lost upon exposure to pronase E, trypsin, amylase, and lipase [18]. The activity was maintained after lysozyme treatment, indicating that the protein might be glycosylated, while treatment with lipase and α-amylase can explain the lack of carbohydrate or lipid moieties. Overall, our data indicated the presence of antimicrobial substances (protein-like) in the bacterial CE of Fa17.2.

#### 3.2.4. Effectiveness of Inhibitory Activity upon Heat, pH, and Detergent Exposure 

**Figure 5:**
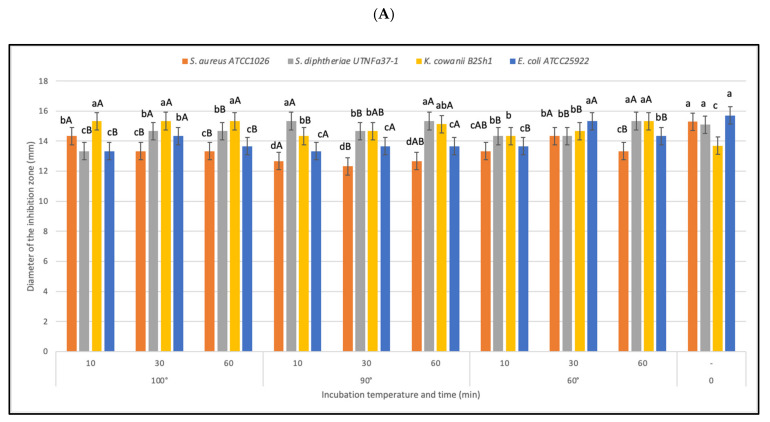

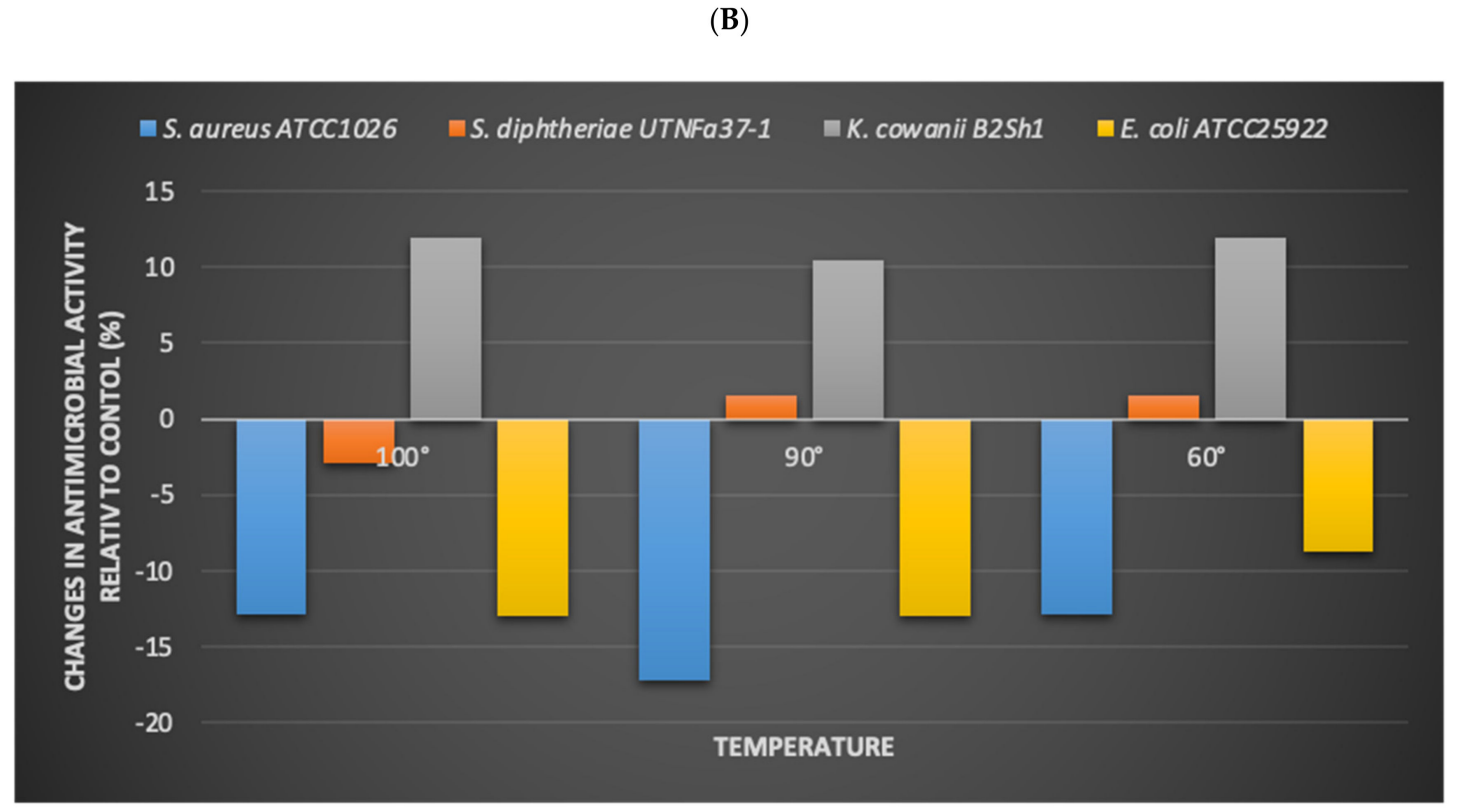
Effect of heat on bacteriocin activity. (**A**) Diameter of the inhibition zone (mm) at different temperatures and incubation time. Bars are the means ± standard error. Values with different letters are significantly different *p* < 0.05. Small letters show the difference between temperature-incubation time and control (LSD with Bonferroni correction); capital letters show the differences within the incubation time (Duncan’s test). (**B**) The influence of pathogen in the inhibitory activity. The changes (%) in antimicrobial activity relative to the control are shown.

**Figure 6:**
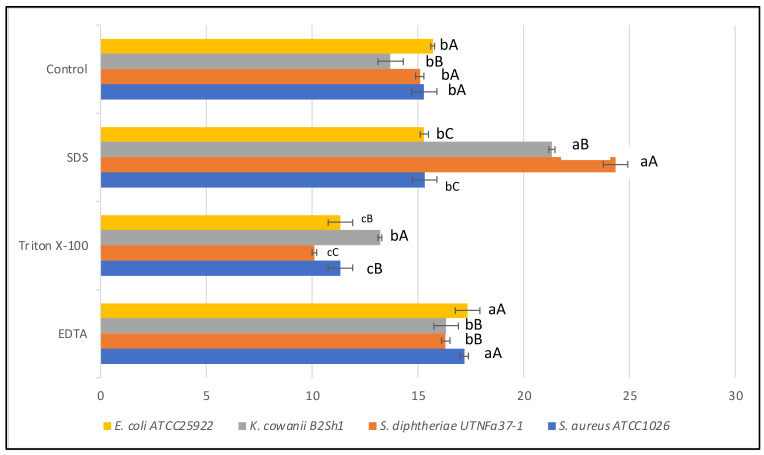
Effect of EDTA, Triton-X100, SDS on antimicrobial activity of CE obtained from Fa17.2. Diameter of the inhibition zone (mm) is shown. Bars are the means ± standard error. Values with different letters are significantly different *p* < 0.05. Small letters show the difference between treatment-pathogen and control (LSD with Bonferroni correction); capital letters indicate the differences within pathogen (Duncan’s test).

The antimicrobial substance was found to be heat stable at all temperatures and times tested, this feature is important when selecting bacteriocinogenic producer strains intended to be used as preservation agents in processed foods. In this study, the statistical analysis revealed that the effectiveness of inhibitory activity was influenced by both temperature-time and pathogen-temperature interactions (Figure 5). Analysis from the split-split-plot design (where main plot: pathogen; sub-plot 1: temperature; and sub-plot 2: incubation time) indicated that the activity was maintained with the incubation time at all four temperatures tested, with a significant increase versus control recorded after 30 and 60 min of incubation (LSD with Bonferroni correction) (Figure 5A). The greatest activity was registered against *K. cowanii* B2Sh1 at all temperature tested indicating that the antimicrobial effectiveness is pathogen-dependent (Figure 5B). Such an increase was not observed in the case of the reference LP strain (data not shown). We hypothesized that the increased inhibitory activity after heat exposure might follow the same path as the thermal process-induced chemical reaction between active elements such as the amino and carbonyl groups, known as the Maillard reaction [42]. Previous studies reported the efficacy of Maillard reaction products with inhibitory action against pathogens, these properties might be linked with the high molecular weight of the proteins released which can bind chemical elements such as iron, copper, or zinc, increasing the antimicrobial effect [43]. Considering the tropical microenvironment origin of the raw material, this might be a significant finding as other studies did not mention such property of heat-time inducing inhibitory activity of *B. subtilis*. At the autoclavation temperature (121 °C for 15 min) the activity was maintained against the four indicator strains under study (data not shown), suggesting the benefit of these molecules if tested as preservatives in association with thermal processing foods. A statistically significant increase (*p* < 0.05) in activity was observed in highly acidic conditions (pH 2.0) towards all indicator strains. Table 2 shows the diameter of the inhibition zone registered against indicator bacteria at different pH treatments of CE. The results indicated that the acidity stimulates the antimicrobial activity, due to the increase in bacteriocin solubility or due to the ability of acids to pass beyond the target cell membranes acidifying the cytoplasm and increasing its permeability [44]. At pH 4.0 and 6.0, the activity was maintained, while a significant decrease was registered at pH 8.0. Nonetheless, the data obtained from CE neutralization only provide a preliminary indication of the active ingredients. Other experiments, integrated CE pH control and acidification of growing cultures, and further investigation is need it to better verify the potential role of organic acids. In agreement with other studies, we suggest that organic acids, if present, may have potentiated the activity of other antimicrobial metabolites, which can trigger acidification and/or acid-mediated cell membrane variation to exert an apparent antagonistic effect [45]. In addition to their pH minimizing characteristics, the antimicrobial effect of organic acids might reflect a specific mode of action that can subjectively be independent of pH. For example, acids permeabilizes the outer membrane of Gram-negative species, causing structural alterations in the phospholipid components [46]. However, the resistance of BLIS to different treatments such as acidity and temperature is an important characteristic of a probiotic strain, as this resistance might enhance the strain capacity to pass through the digestive tract, adhere to, and colonize the host gut [47]. In addition, a significant increase in antimicrobial activity (*p* < 0.05) relative to the untreated counterpart was observed when adding EDTA and SDS for both strains (Figure 6)**.** The positive effect of EDTA and SDS on the inhibitory activity against Gram-negative species was previously described [48]. This activity was linked to the increase in outer membrane permeability beyond extracting cations (Ca^2+^, Mg^2+^); thus, allowing bacteriocins to reach the cytoplasmic membrane. Similarly, in this study, the effectiveness of inhibitory activity was positively influenced by the treatment with SDS and chelating EDTA agent. Likewise, a slight decrease in activity was observed when CE was treated with Triton-X100. Comparable results were obtained with the reference to the LP strain (data not shown). In a similar study, no such increase in activity was observed when treating the cell-free supernatant from *B. subtilis* RLID 12.1 with EDTA, Triton-X100, or SDS, indicating that such effect might be bacteriocin-dependent [18]. In conclusion, our results indicated that the efficiency of bacteriocin-like substances of the selected Fa17.2 strain was positively regulated by heat, acidic condition, and chelating agents, these features might help for further identification of the mode of action against multidrug-resistant pathogens. 

### 3.3. Molecular Weight Estimation of BLIS Substances

**Figure 7:**
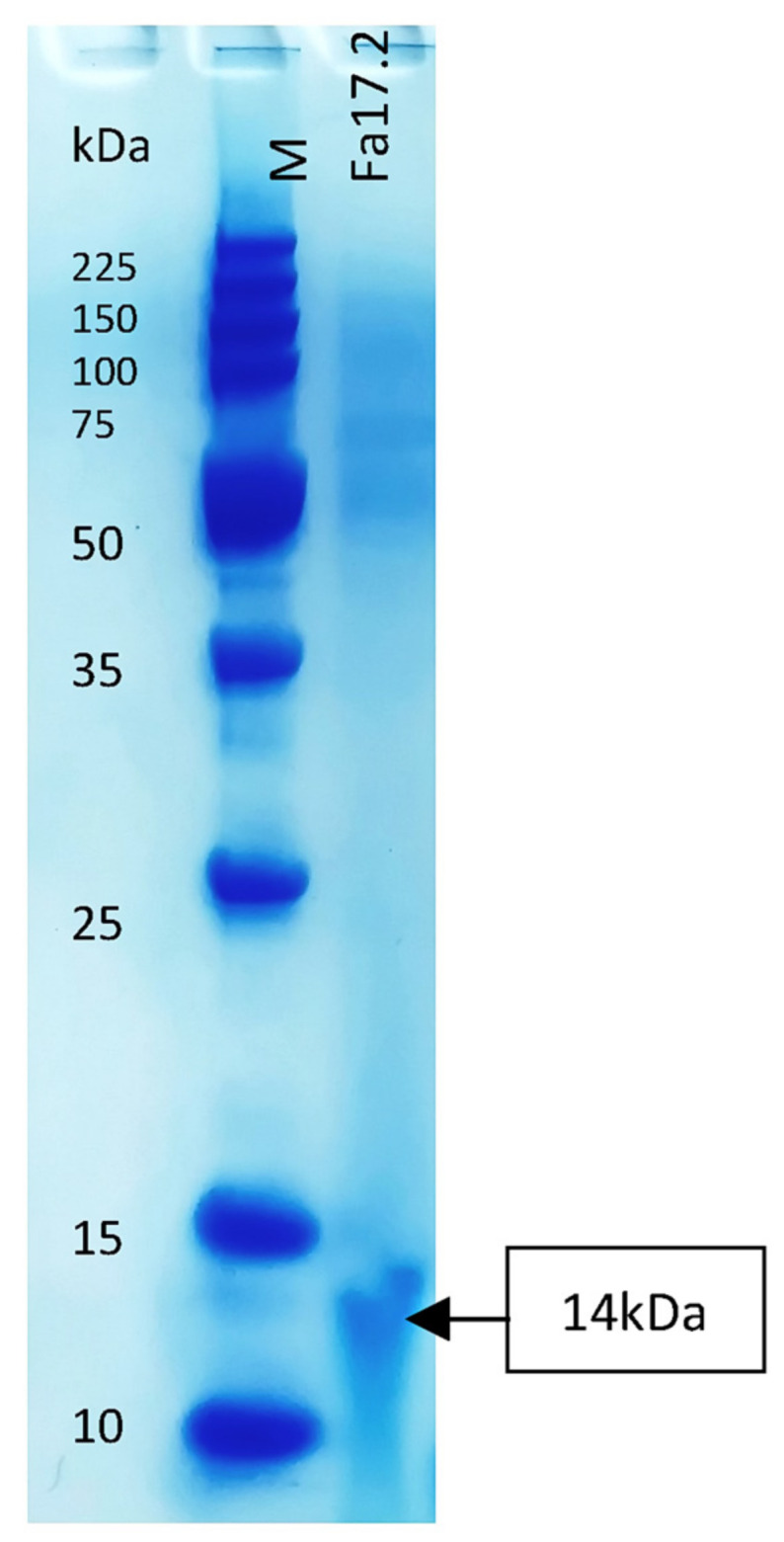
Tricine-SDS-PAGE of the partial purified bacteriocin of Fa17.2. Legend: M: molecular marker (low molecular range marker Promega); Fa17.2 purified peptide extract from *B. subtilis* Fa17.2 strain.

Members of the genus *Bacillus* are known to produce a wide arsenal of antimicrobial substances, including peptide and lipopeptide antibiotics, and bacteriocins [49]. Some bacteriocins were earlier characterized [50]. The molecular weight of the band in Tricine-SDS-PAGE was estimated to be about 14 kDa (Figure 7). The size was larger than previously characterized *B. subtilis* L-Q11 of 3.5 kDa [51] and 5 kDa of *B. subtilis* RLID 12.1 [18]. Bacteriocins with antimicrobial activity, greater than 10 kDa of class III, were already detected in different *Bacillus* species [49]. For example, baciamin, bacisubin, CAMT2, and Bac14B showed high antifungal and antimicrobial activity [52,53,54,55]. Further biochemical and molecular characterization after complete purification will be undertaken. 

## 4. Conclusions

To the best of our knowledge, this is the first study showing the presence of *B. subtilis* in tropical *Bromelia* sp. inflorescence showing strain-specific probiotic and antimicrobial strength. The Fa17.2 strain exerted high tolerance to artificial gastric acid, bile, and sodium chloride and was sensible to various antibiotics. The selected strain tolerates high temperatures thus, unlike many other probiotic strains can resist food processing. To a lesser extent, the results were superior to the probiotic LA strain. The Fa17.2 strain was considered safe as no hemolysis was detected in sheep blood agar. In addition, it generates highly thermostable antimicrobials with characteristics very similar to the bacteriocinogenic LP strain. The CE produced by the selected strain was effective against several indicator microorganisms including Gram-negative bacteria, thus using CE as antimicrobial components can be an interesting plan to combat spoilage microorganisms in foods. The antimicrobial activity was stimulated by heat and remains active over a wide pH range. The molecular weight of the partially purified BLIS was about 14 kDa, but more research is required to determine its chemical composition. The present characterization revealed interesting properties of *B. subtilis* strain Fa17.2 with potential applications for biological control of pathogenic strains. Additional research should be aimed at identifying the molecular mechanism of pathogen inhibition. Similarly, the selected microbiome associated with such a microenvironment (tropical flowers and fruits) must be further tailored as a unique probiotic consortium inoculum harboring several species that can confer multifunctional characteristics on the raw matrix with which they interact. Taken together, the data obtained from this research represent the starting point of a demanding study aimed to select beneficial microorganisms from native raw materials that should be further exploited in the food market as an innovative strategy to maintain or improve the quality of the products, guaranteeing food security in the region, while at the same time implementing sustainable solutions in developing countries.

## Figures and Tables

**Table 1:** Effect of enzymes on the antimicrobial activity.

Strains	Indicator Strains	NCE + Enzymes (1 mg/mL)					NCE (Control)
		α Chymotrypsin	Lysozyme	Proteinase K	Catalase	Pepsin	
Fa17.2	*S. aureus* ATCC1026	9.33 ± 0.1 ^b^	10.33 ± 0.1 ^a^	6.01 ± 0.1 ^c^	9.33 ± 0.2 ^b^	9.67 ± 0.2 ^b^	10.33 ± 0.1 ^a^
	*S.dysenteriae* UTNFa37-1	9.67 ± 0.6 ^b^	10.33 ± 0.1 ^a^	6.01 ± 0.1 ^b^	9.33 ± 0.2 ^b^	9.67 ± 0.2 ^b^	10.33 ± 0.1 ^a^
	*K. cowanii* B2Sh1	8.33 ± 0.2 ^c^	11.20 ± 0.2 ^a^	6.01 ± 0.1 ^d^	9.33 ± 0.2 ^b^	8.67 ± 0.2 ^bc^	11.33 ± 0.2 ^a^
	*E. coli* ATCC25922	9.33 ± 0.4 ^b^	10.67 ± 0.1 ^a^	6.01 ± 0.1 ^c^	9.33 ± 0.2 ^b^	9.33 ± 0.2 ^b^	10.67 ± 0.1 ^a^
LP	*S. aureus* ATCC1026	9.67 ± 0.2 ^b^	10.33 ± 0.2 ^a^	6.01 ± 0.1 ^d^	8.33 ± 0.2 ^c^	9.67 ± 0.1 ^b^	10.33 ± 0.2 ^a^
	*S.dysenteriae* UTNFa37-1	8.67 ± 0.6 ^bc^	10.33 ± 0.2 ^a^	6.01 ± 0.1 ^d^	9.33 ± 0.2 ^b^	8.33 ± 0.2 ^c^	10.33 ± 0.2 ^a^
	*K. cowanii* B2Sh1	8.33 ± 0.2 ^b^	9.67 ± 0.2 ^a^	6.01 ± 0.1 ^c^	8.67 ± 0.1 ^ab^	8.33 ± 0.2 ^b^	9.67 ± 0.2 ^a^
	*E. coli* ATCC25922	8.33 ± 0.2 ^b^	10.33 ± 0.2 ^a^	6.01 ± 0.1 ^c^	8.67 ± 0.1 ^b^	8.67 ± 0.1 ^b^	10.33 ± 0.2 ^a^

Data are mean ± standard error. Values in the same row with small letters are significantly different versus NCE (*p* < 0.05); NCE (control): neutralized CE (10 min heat at 80 °C and pH 6.0); CE: crude-extract.

**Table 2:** Antimicrobial activity (inhibition zone expressed in mm) of the CE at different pH against indicator bacteria.

Samples	Indicator Strains	Diameter of the Inhibition Zone (mm)				
		pH				Control CE (No Treatment)
		2.0	4.0	6.0	8.0	
Fa17.2	*S. aureus* ATCC1026	16.1 ± 0.1 ^eA^	14.6 ± 1.0 ^dC^	9.3 ± 1.0 ^abD^	8.1 ± 0.1 ^bE^	15.3 ± 0.6^B^
	*S. dysenteriae* UTNFa37-1	18.3 ± 0.6 ^cA^	15.1 ± 0.1 ^cdB^	9.1 ± 0.1 ^bC^	8.1 ± 0.1 ^bD^	15.1 ± 0.2 ^B^
	*K. cowanii* B2Sh1	16.1 ± 0.1 ^eA^	14.6 ± 1.0 ^dB^	9.1 ± 0.1 ^bD^	8.1 ± 0.1 ^bE^	13.7 ± 0.6 ^C^
	*E. coli* ATCC25922	15.3 ± 0.6 ^fA^	14.1 ± 0.1 ^dB^	9.7 ± 1.0 ^aD^	9.1 ± 0.1 ^abD^	13.3 ± 0.1 ^C^
LP	*S. aureus* ATCC1026	17.1 ± 0.1 ^dA^	16.1 ± 0.1 ^bB^	9.7 ± 1.0 ^aC^	9.3 ± 0.6 ^aC^	15.7 ± 0.6 ^B^
	*S. dysenteriae* UTNFa37-1	26.1 ± 0.6 ^aA^	17.3 ± 1.0 ^aB^	9.7 ± 1.0 ^aD^	8.1 ± 0.1 ^bE^	16.1 ± 0.1 ^C^
	*K. cowanii* B2Sh1	20.7 ± 0.6 ^bA^	15.3 ± 1.0 ^cB^	9.1 ± 0.1 ^bC^	8.1 ± 0.1 ^bD^	15.1 ± 0.1 ^B^
	*E. coli* ATCC25922	21.7 ± 1.0 ^bA^	15.3 ± 0.1 ^cB^	9.1 ± 0.1 ^bD^	7.1 ± 0.1 ^cE^	12.3 ± 0.6 ^C^

Data are mean ± standard error. Values in the same column with small different letters are statistically different (*p* < 0.05). Values in the same row with capital letters are significantly different versus control (no treatment).

## Data Availability

Not applicable.

## Supplementary Materials

The following are available online at: https://www.mdpi.com/article/10.3390/microorganisms10050860/s1, Table S1. Cell viability (log CFU/mL) registered during incubation with the gastric juice at different pH and 0.3% bile (w/v, oxgall). Table S2. The cell viability (log CFU/mL) was registered during incubation with NaCl at different concentrations and temperatures for 24 h. Table S3. Antibiotic susceptibility of the B. subtilis Fa17.2 Table S4. Diameter of the zone of inhibition (mm) produced by CE of Fa17.2 and LP in different media against S. aureus ATCC1026. Table S5. Diameter of the zone of inhibition (mm) produced by CE of Fa17.2 and LP in different media against E. coli ATCC25922. Table S6. Diameter of the zone of inhibition (mm) produced by CE of Fa17.2 and LP in different media enriched with different carbon sources against K. cowanii B2Sh1. Table S7. Diameter of the zone of inhibition (mm) produced by CE of Fa17.2 and LP in different media against S. dysenteriae UTNFa37-1.

## References

[1] Prestinaci F., Pezzotti P., Pantosti A. (2015). Antimicrobial resistance: A global multifaceted phenomenon. Pathog. Glob. Health.

[2] Ministry of Public Health, Ecuador SIVE Surveillance Subsystem—Water and Foodborne Diseases Alert Ecuador, 2021, SE 18. https://www.salud.gob.ec/wp-content/uploads/2021/05/Etas-SE-18.pdf.

[3] Tenea G.N., Olmedo D. (2021). Antimicrobial cocktail combining specific peptide extracts from native probiotic bacteria hamper adulteration of ready-to-eat mango wedges. Appl. Sci..

[4] FAO, WHO (2006). Probiotics in Food: Health and Nutritional Properties and Guidelines for Evaluation.

[5] Capurso L. (2019). Thirty years of *Lactobacillus rhamnosus* GG: A Review. J. Clin. Gastroenterol..

[6] Park K.M., Yoon S.G., Choi T.H., Kim H.J., Park K.J., Koo M. (2020). The bactericidal effect of a combination of food-grade compounds and their application as alternative antibacterial agents for food contact surfaces. Foods.

[7] Kimelman H., Shemesh M. (2019). Probiotic bifunctionality of *Bacillus subtilis*—Rescuing lactic acid bacteria from desiccation and antagonizing pathogenic *Staphylococcus aureus*. Microorganisms.

[8] Urdaci M.C., Pinchuk I. (2004). Antimicrobial activity of *Bacillus* probiotics. Bacterial Spore Formers–Probiotics and Emerging Applications.

[9] Su Y., Liu C., Fang H., Zhang D. (2020). Bacillus subtilis: A universal cell factory for industry, agriculture, biomaterials and medicine. Microb. Cell Fact..

[10] Kim B.J., Hong J.H., Jeong Y.S., Jung H.K. (2014). Evaluation of two *Bacillus subtilis* strains isolated from Korean fermented food as probiotics against loperamide-induced constipation in mice. J. Korean Soc. Appl. Biol. Chem..

[11] Lu Z., Guo W., Liu C. (2018). Isolation, identification and characterization of novel *Bacillus subtilis*. J. Vet. Med. Sci..

[12] Luo X.Z., Shi B.H., Zheng H., Wei-Bin W.U., Shi Q.Q. (2008). Physical and chemical properties and antimicrobial spectrum of subtilin from *Bacillus subtilis* FB123. J. Microbiol..

[13] Foster L.M., Tompkins T.A., Dahl W.J. (2011). A comprehensive post-market review of studies on a probiotic product containing *Lactobacillus helveticus* R0052 and *Lactobacillus rhamnosus* R0011. Benef. Microb..

[14] Bernardeau M., Lehtinen M.J., Forssten S.D., Nurminen P. (2017). Importance of the gastrointestinal life cycle of *Bacillus* for probiotic functionality. J. Food Sci. Technol..

[15] Cutting S.M. (2011). *Bacillus* probiotics. Food Microbiol..

[16] Ilinskaya O.N., Ulyanova V.V., Yarullina D.R., Gataullin I.G. (2017). Secretome of intestinal *Bacilli*: A natural guard against pathologies. Front Microbiol..

[17] Lefevre M., Racedo S.M., Denayrolles M., Ripert G., Desfougères T., Lobach A.R., Simon R., Pélerin F., Jüsten P., Urdaci M.C. (2017). Safety assessment of *Bacillus subtilis* CU1 for use as a probiotic in humans. Regul. Toxicol. Pharmacol..

[18] Ramachandran R., Chalasani A.G., Lal R., Roy U. (2014). A broad-spectrum antimicrobial activity of *Bacillus subtilis* RLID12.1. Sci. World J..

[19] Stein T. (2005). *Bacillus subtilis* antibiotics: Structures, syntheses and specific functions. Mol. Microbiol..

[20] (2021). Commercial Bulletin. Ministry of Production, Foreign Trade, Investment and Fisheries 2021, Twelfth Edition. https://www.produccion.gob.ec/wp-content/uploads/2021/12/VFBoletinComercioExteriorDiciembre2021-final.pdf?fbclid=IwAR3dpQeWuwUgQF8IOVsb7SSmLCbMPBHZkKhpxXqMA7HPEY61wWhN.

[21] Benavidez A.B., Ulcuango M., Yepez L., Tenea G.N. (2016). Assessment of the in vitro bioactive properties of lactic acid bacteria isolated from native ecological niches of Ecuador. Rev. Arg. Microbiol..

[22] Corcoran B.M., Stanton C., Fitzgerald G.F., Ross R.P. (2005). Survival of probiotic lactobacilli in acidic environments is enhanced in the presence of metabolizable sugars. Appl. Environ. Microbiol..

[23] Yasmin I., Saeed M., Khan W.A., Khaliq A., Chughtai M., Iqbal R., Tehseen S., Naz S., Liaqat A., Mehmood T. (2020). In vitro probiotic potential and safety evaluation (hemolytic, cytotoxic activity) of *Bifidobacterium* strains isolated from raw camel milk. Microorganisms.

[24] EFSA (2012). Panel on additives and products or substances used in animal feeds. Guidance on the assessment of bacterial susceptibility to antimicrobials of human and veterinary importance. EFSA J..

[25] Garzon K., Ortega C., Tenea G.N. (2017). Characterization of bacteriocin-producing lactic acid bacteria isolated from native fruits of Ecuadorian Amazon. Pol. J. Microbiol..

[26] Waterhouse A.M., Procter J.B., Martin D.M., Clamp M., Barton G.J. (2009). Jalview version 2-a multiple sequence alignment editor and analysis workbench. Bioinformatics.

[27] Lee N.K., Han K.J., Son S.H., Eom S.J., Lee S.K., Paik H.D. (2015). Multifunctional effect of probiotic *Lactococcus lactis* KC24 isolated from kimchi. LWT-Food Sci. Technol..

[28] Patel A.K., Ahire J.J., Pawar S.P., Chaudhari B.L., Chincholkar S.B. (2009). Comparative accounts of probiotic characteristics of *Bacillus* spp. isolated from food wastes. Food Res. Int..

[29] Jeon H.L., Lee N.K., Yang S.J., Kim W.S., Paik H.D. (2017). Probiotic characterization of *Bacillus subtilis* P223 isolated from kimchi. Food Sci. Biotech..

[30] Bevilacqua A., Altieri C., Carbo M.R., Sinigaglia M., Ouoba L.I.I. (2010). Characterization of lactic acid bacteria isolated from Italian Belladi Cerignola table olives: Selection of potential multifunctional starter cultures. J. Food Sci..

[31] Gueimonde M., Sánchez B.G., de Los Reyes-Gavilán C., Margolles A. (2013). Antibiotic resistance in probiotic bacteria. Front. Microbiol..

[32] Lee N.K., Kim W.S., Paik H.D. (2019). *Bacillus* strains as human probiotics: Characterization, safety, microbiome, and probiotic carrier. Food Sci. Biotechnol..

[33] Sarkar P.K., Hasenack B., Nout M.J. (2002). Diversity and functionality of *Bacillus* and related genera isolated from spontaneously fermented soybeans (Indian Kinema) and locust beans (African Soumbala). Int. J. Food Microbiol..

[34] Hong H.A., Duc L.H., Cutting S.M. (2005). The use of bacterial spore formers as probiotics. FEMS Microbiol. Rev..

[35] EFSA (2012). Scientific opinion on the maintenance of the list of QPS biological agents intentionally added to food and feed. EFSA J..

[36] Khardziani T., Kachlishvili E., Sokhadze K., Elisashvili V., Weeks R., Chikindas M.L., Chistyakov V. (2017). Elucidation of *Bacillus subtilis* KATMIRA 1933 potential for spore production in submerged fermentation of plant raw materials. Probiotics Antimicrob. Proteins.

[37] Monteiro S.M., Clemente J.J., Carrondo M.J., Cunha A. (2014). Enhanced spore production of *Bacillus subtilis* grown in a chemically defined medium. Ai Mag..

[38] Khochamit N., Siripornadulsil S., Sukon P., Siripornadulsil W. (2015). Antibacterial activity and genotypic-phenotypic characteristics of bacteriocin-producing *Bacillus subtilis* KKU213: Potential as a probiotic strain. Microbiol. Res..

[39] Xie J., Zhang R., Shang C., Guo Y. (2009). Isolation and characterization of a bacteriocin produced by an isolated *Bacillus subtilis* LFB112 that exhibits antimicrobial activity against domestic animal pathogens. Afr. J. Biotechnol..

[40] Yi Y., Zhang Z., Zhao F., Liu H., Yu L., Zha J., Wang G. (2018). Probiotic potential of *Bacillus velezensis* JW: Antimicrobial activity against fish pathogenic bacteria and immune enhancement effects on *Carassius auratus*. Fish Shellfish Immunol..

[41] Sharma A., Satyanarayana T. (2013). Comparative genomics of *Bacillus* species and its relevance in industrial microbiology. Genom. Insights.

[42] Hiramoto S., Itoh K., Shizuuchi S., Kawachi Y., Morishita Y., Nagase M., Suzuki Y., Nobuta Y., Sudou Y., Nakamura O. (2004). Melanoidin, a food protein-derived advanced Maillard reaction product, suppresses *Helicobacter pylori* in vitro and in vivo. Helicobacter.

[43] Kukuminato S., Koyama K., Koseki S. (2021). Antibacterial properties of melanoidins produced from various combinations of Maillard reaction against pathogenic bacteria. Microbiol. Spectr..

[44] Banerjee S.P., Dora K.C., Chowdhury S. (2013). Detection, partial purification and characterization of bacteriocin produced by *Lactobacillus brevis* FPTLB3 isolated from freshwater fish: Bacteriocin from *Lb. brevis* FPTLB3. J. Food Sci. Technol..

[45] Arena M.P., Silvain A., Normanno G., Grieco F., Drider D., Spano G., Fiocco D. (2016). Use of *Lactobacillus plantarum* strains as a bio-control strategy against food-borne pathogenic microorganisms. Front. Microbiol..

[46] Alakomi H.L., Skyttä E., Saarela M., Mattila-Sandholm T., Latva-Kala K., Helander I.M. (2000). Lactic acid permeabilizes gram-negative bacteria by disrupting the outer membrane. Appl. Environ. Microbiol..

[47] Goyal C., Malik R.K., Pradhan D. (2018). Purification and characterization of a broad spectrum bacteriocin produced by a selected *Lactococcus lactis* strain 63 isolated from Indian dairy products. J. Food Sci. Technol..

[48] Gálvez A., Abriouel H., López R.L., Ben Omar N. (2007). Bacteriocin-based strategies for food biopreservation. Int. J. Food Microbiol..

[49] Abriouel H., Franz C.M., Ben Omar N., Gálvez A. (2011). Diversity and applications of *Bacillus* bacteriocins. FEMS Microbiol. Rev..

[50] Fuchs S.W., Jaskolla T.W., Bochmann S., Kötter P., Wichelhaus T., Karas M., Stein T., Entian K.D. (2011). Entianin, a novel subtilin-like lantibiotic from *Bacillus subtilis* subsp*. spizizenii* DSM 15029T with high antimicrobial activity. Appl. Environ. Microbiol..

[51] Qin Y., Wang Y., He Y., Zhang Y., She Q., Chai Y., Li P., Shang Q. (2019). Characterization of subtilin L-Q11, a novel class I bacteriocin synthesized by *Bacillus subtilis* L-Q11 isolated from orchard soil. Front. Microbiol..

[52] Hammami I., Jaouadi B., Bacha A.B., Rebai A., Bejar S., Nesme X., Rhouma A. (2012). *Bacillus subtilis* bacteriocin Bac 14B with a broad inhibitory spectrum: Purification, amino acid sequence analysis, and physicochemical characterization. Biotechnol. Bioprocess Eng..

[53] Wong J.H., Hao J., Cao Z., Qiao M., Xu H., Bai Y., Ng T.B. (2008). An antifungal protein from *Bacillus amyloliquefaciens*. J. Appl. Microbiol..

[54] An J., Zhu W., Liu Y., Zhang X., Sun L., Hong P., Wang I., Xu C., Xu D., Liu H. (2015). Purification and characterization of a novel bacteriocin CAMT2 produced by *Bacillus amyloliquefacien*s isolated from marine fish *Epinephelus areolatus*. Food Control..

[55] Liu Y., Chen Z., Ng T.B., Zhang J., Zhou M., Song F., Lu F., Liu Y. (2007). Bacisubin, an antifungal protein with ribonuclease and hemagglutinating activities from *Bacillus subtilis* strain B-916. Peptides.

