# Supplementary material for: Probiotic Characteristics and Antimicrobial Potential of a Native Bacillus subtilis Strain Fa17.2 Rescued from Wild Bromelia sp. Flowers
Source: Microorganisms. 2022 Apr 21;10(5):860. doi: 10.3390/microorganisms10050860 (PMC9145066; doi:10.3390/microorganisms10050860)
Supplement: Supplementary file 1 [file microorganisms-10-00860-s001.zip › Table S1.pdf]

Table S1. Cell viability (logCFU/ml) registered during incubation with the gastric juice at different pH and 0.3% bile (oxgall w/v).

pH	Time (h)	Cell viability (logCFU/ml)	
		**Fa17.2**	**LA**
**2.5**	**0**	8.24 ± 0.03	8.25 ± 0.01
	**1**	8.27 ± 0.01	8.10 ± 0.02
	**3**	7.81± 0.04	7.68± 0.04
	**4**	7.72± 0.06	7.04± 0.06
**3**	**0**	8.22 ± 0.01	8.28 ± 0.01
	**1**	8.28 ± 0.05	8.12 ± 0.03
	**3**	7.83± 0.01	7.46± 0.01
	**4**	7.75± 0.04	7.25± 0.13
**3.5**	**0**	8.24 ± 0.02	8.25 ± 0.02
	**1**	8.33± 0.03	8.14± 0.01
	**3**	7.89± 0.01	7.70± 0.02
	**4**	7.68± 0.01	7.28± 0.02
0.3% bile (oxgall, w/v)	**0**	8.29 ± 0.06	8.30 ± 0.03
	**1**	8.75± 0.04	8.09± 0.01
	**3**	8.89± 0.05	7.30± 0.04
	**4**	9.04± 0.04	6.86± 0.06

Data represent the mean ± standard error.
