# Supplementary material for: Probiotic Characteristics and Antimicrobial Potential of a Native Bacillus subtilis Strain Fa17.2 Rescued from Wild Bromelia sp. Flowers
Source: Microorganisms. 2022 Apr 21;10(5):860. doi: 10.3390/microorganisms10050860 (PMC9145066; doi:10.3390/microorganisms10050860)
Supplement: Supplementary file 1 [file microorganisms-10-00860-s001.zip › Table S2.pdf]

Table S2. The cell viability (logCFU/ml) was registered during incubation with NaCl at different concentrations and temperatures for 24 hours.

Temperature	NaCl (%)	Time (h)	Cell viability (logCFU/ml)	
			Fa17.2	LA
15 °C	1	0	8.18 ± 0.01	8.20 ± 0.06
		24	7.98 ± 0.05	7.80 ± 0.04
	3	0	8.18 ± 0.01	8.20 ± 0.06
		24	7.82± 0.05	7.65± 0.02
	5	0	8.18 ± 0.01	8.20 ± 0.06
		24	7.62± 0.02	6.90± 0.04
45 °C	1	0	8.18 ± 0.01	8.20 ± 0.06
		24	7.93± 0.03	7.90± 0.02
	3	0	8.18 ± 0.01	8.20 ± 0.06
		24	7.54± 0.05	7.89± 0.13
	5	0	8.18 ± 0.01	8.20 ± 0.06
		24	7.45± 0.05	7.49± 0.05

Data represent the mean ± standard error.
