# Supplementary material for: Probiotic Characteristics and Antimicrobial Potential of a Native Bacillus subtilis Strain Fa17.2 Rescued from Wild Bromelia sp. Flowers
Source: Microorganisms. 2022 Apr 21;10(5):860. doi: 10.3390/microorganisms10050860 (PMC9145066; doi:10.3390/microorganisms10050860)
Supplement: Supplementary file 1 [file microorganisms-10-00860-s001.zip › Table S3.pdf]

Table S3. Antibiotic susceptibility of the *B. subtilis* Fa17.2

**Antimicrobial E-test strip**	**MIC (mg/L)**	**Susceptibility***	**EFSA cut-off values (mg/L) *Bacillus* spp.**
Amoxicillin	0.75	S	Nd
Amoxicillin: clavulanic acid	0.04	S	4
Ampicillin	0.06	S	1
Cefotaxime	1	S	4
Kanamycin	2	S	4
Gentamycin	0.75	S	4
Tetracycline	0.13	S	8

Nd: not determined

Susceptible (S): a bacterial strain is defined as susceptible when it is inhibited at a concentration of a specific antimicrobial equal to or lower than the established cut-off value (S ≤ x mg / L). Resistant (R): a bacterial strain is defined as resistant when it is not inhibited at a concentration of a specific antimicrobial above the established cut-off value (R> x mg / L). ** MIC reference for *L. plantarum.*
