# Supplementary material for: Probiotic Characteristics and Antimicrobial Potential of a Native Bacillus subtilis Strain Fa17.2 Rescued from Wild Bromelia sp. Flowers
Source: Microorganisms. 2022 Apr 21;10(5):860. doi: 10.3390/microorganisms10050860 (PMC9145066; doi:10.3390/microorganisms10050860)
Supplement: Supplementary file 1 [file microorganisms-10-00860-s001.zip › Table S4.pdf]

Table S4. Diameter of the zone of inhibition (mm) produced by CE of Fa17.2 and LP in different media against *S. aureus* ATCC1026. Groups of measurements in the same column followed by different small letter are statistically different ($p < 0.05$).

Medium	Concentration of carbohydrate	Average of the diameter of the inhibition zone (mm)	
		**Fa17.2**	**LP**
MRS+ Glycerol (MRSGly)	5%	13.1 ± 0.2^{d}	13.1 ± 0.2^{de}
	10%	13.1 ± 0.2^{d}	13.6 ± 0.5^{d}
	20%	14.1 ± 0.2^{c}	17.1 ± 0.2^{a}
	40%	13.1 ± 0.2^{d}	11.6 ± 0.5^{f}
	50%	10.3 ± 0.5^{f}	12.1 ± 0.2^{e}
MRS+ Sucrose (MRSS)	5%	13.1 ± 0.2^{d}	15.1 ± 0.2^{b}
	10%	13.6 ± 0.5^{cd}	14.3 ± 0.5^{c}
	20%	14.1 ± 0.2^{c}	13.3 ± 0.5^{d}
	40%	13.1 ± 0.2^{cd}	13.3 ± 0.5^{d}
	50%	13.1 ± 0.2^{cd}	12.1 ± 0.2^{e}
MRS+ Glucose (MRSG)	5%	13.3 ± 0.5^{cd}	14.3 ± 0.5^{c}
	10%	16.1 ± 0.2^{a}	14.3 ± 0.5^{c}
	20%	16.1 ± 0.2^{a}	14.1 ± 0.2^{c}
	40%	13.1 ± 0.2^{d}	12.6 ± 0.1^{e}
	50%	13.1 ± 0.2^{d}	12.1 ± 0.2^{e}
Control MRS (no treatment)	None	11.3 ± 0.5^{e}	11.3 ± 0.5^{f}

Data represent the mean ± standard error.
