# Supplementary material for: Probiotic Characteristics and Antimicrobial Potential of a Native Bacillus subtilis Strain Fa17.2 Rescued from Wild Bromelia sp. Flowers
Source: Microorganisms. 2022 Apr 21;10(5):860. doi: 10.3390/microorganisms10050860 (PMC9145066; doi:10.3390/microorganisms10050860)
Supplement: Supplementary file 1 [file microorganisms-10-00860-s001.zip › Table S5.pdf]

Table S5. Diameter of the zone of inhibition (mm) produced by CE of Fa17.2 and LP in different media against *E. coli* ATCC25922. Groups of measurements in the same column followed by different small letter are statistically different ($p < 0.05$).

Medium	Concentration of carbohydrate	Average of the diameter of the inhibition zone (mm)	
		Fa17.2	LP
MRS+ Glycerol (MRSGly)	5%	14.3 ± 0.5[ab]	14.3 ±0.5[b]
	10%	14.1 ± 0.1[b]	12.3 ± 0.2[d]
	20%	13.6 ± 0.5[c]	15.3 ± 0.2[a]
	40%	12.6 ± 0.5[de]	13.1 ± 0.2[cd]
	50%	13.3 ± 0.5[cd]	12.3 ± 0.5[d]
MRS+ Sucrose (MRSS)	5%	14.1 ± 0.2[b]	13.1 ± 0.2[cd]
	10%	14.3 ± 0.5[ab]	12.3 ± 0.5[d]
	20%	14.1 ± 0.2[b]	13.3 ± 0.2[c]
	40%	12.3 ± 0.5[d]	12.3 ± 0.5[d]
	50%	12.3 ± 0.5[d]	12.1 ± 0.2[e]
MRS+ Glucose (MRSG)	5%	14.1 ± 0.1[b]	13.3 ± 0.5[c]
	10%	15.3 ± 0.2[a]	13.3 ± 0.2[c]
	20%	15.1 ± 0.2[a]	14.1 ± 0.2[b]
	40%	12.3 ± 0.5[d]	13.3 ± 0.5[c]
	50%	12.6 ± 0.5[d]	12.3 ± 0.5[d]
Control MRS (no treatment)	none	13.3 ± 0.5[c]	12.3 ± 0.20[d]

Data represent the mean ± standard error.
