# Supplementary material for: Probiotic Characteristics and Antimicrobial Potential of a Native Bacillus subtilis Strain Fa17.2 Rescued from Wild Bromelia sp. Flowers
Source: Microorganisms. 2022 Apr 21;10(5):860. doi: 10.3390/microorganisms10050860 (PMC9145066; doi:10.3390/microorganisms10050860)
Supplement: Supplementary file 1 [file microorganisms-10-00860-s001.zip › Table S6.pdf]

Table S6. Diameter of the zone of inhibition (mm) produced by CE of Fa17.2 and LP in different media enriched with different carbon sources against *K. cowanii B2Sh1*. Groups of measurements in the same column followed by different small letter are statistically different ($p < 0.05$).

**Medium**	**Concentration of carbohydrate**	**Average of the diameter of the inhibition zone (mm)**	
		**Fa17.2**	**LP**
MRS+ Glycerol (MRSGly)	5%	13.6 ± 0.2[c]	14.1 ± 0.2[c]
	10%	12.3 ± 0.2[cd]	13.6 ± 0.2[cd]
	20%	14.6 ± 0.2[ab]	16.3 ± 0.2[a]
	40%	11.1 ± 0.2[e]	14.1 ± 0.2[c]
	50%	10.2 ± 0.2[f]	12.3 ± 0.2[de]
MRS+ Sucrose (MRSS)	5%	13.6 ± 0.2[c]	14.1 ± 0.2[c]
	10%	14.1 ± 0.2[b]	12.1 ± 0.2[e]
	20%	12.6 ± 0.2[d]	13.3 ± 0.2[de]
	40%	12.3 ± 0.2[cd]	14.1 ± 0.2[c]
	50%	11.6 ± 0.2[de]	12.1 ± 0.2
MRS+ Glucose (MRSG)	5%	11.3 ± 0.2[de]	14.1 ± 0.2[c]
	10%	15.3 ± 0.2[a]	13.1 ± 0.2[d]
	20%	13.6 ± 0.2[c]	14.1 ± 0.2[c]
	40%	12.3 ± 0.2[cd]	13.6 ± 0.5[cd]
	50%	14.6 ± 0.2[ab]	15.1 ± 0.2[b]
Control MRS (no treatment)	None	13.3 ± 0.5[c]	12.6 ± 0.5[de]

Data represent the mean ± standard error.
