# Supplementary material for: Probiotic Characteristics and Antimicrobial Potential of a Native Bacillus subtilis Strain Fa17.2 Rescued from Wild Bromelia sp. Flowers
Source: Microorganisms. 2022 Apr 21;10(5):860. doi: 10.3390/microorganisms10050860 (PMC9145066; doi:10.3390/microorganisms10050860)
Supplement: Supplementary file 1 [file microorganisms-10-00860-s001.zip › Table S7.pdf]

Table S7. Diameter of the zone of inhibition (mm) produced by CE of Fa17.2 and LP in different media against *S. dysenteriae* UTNFa37-1. Groups of measurements in the same column followed by different small letter are statistically different ($p < 0.05$).

Medium	Concentration of carbohydrate	Average of the diameter of the inhibition zone (mm)	
		Fa17.2	LP
MRS+ Glycerol	5%	10.6 ± 0.5^{g}	14.6 ± 0.5^{bc}
	10%	12.3 ± 0.5^{de}	10.6 ± 0.5^{f}
	20%	13.1 ± 0.1^{d}	16.6 ± 0.1^{a}
	40%	12.1 ± 0.1^{e}	13.1 ± 0.1^{d}
	50%	12.1 ± 0.1^{e}	12.3 ± 0.5^{de}
MRS+ Sucrose	5%	14.1 ± 0.1^{c}	13.1 ± 0.1^{d}
	10%	15.1 ± 0.1^{b}	14.3 ± 0.5^{bc}
	20%	13.1 ± 0.1^{d}	13.1 ± 0.1^{d}
	40%	11.1 ± 0.1^{f}	14.1 ± 0.1^{c}
	50%	12.1 ± 0.1^{e}	12.6 ± 0.5^{de}
MRS+ Glucose	5%	12.3 ± 0.5^{de}	12.1 ± 0.1^{e}
	10%	16.3 ± 0.5^{a}	13.3 ± 0.5^{cd}
	20%	16.1 ± 0.1^{a}	12.1 ± 0.1^{e}
	40%	11.3 ± 0.5^{df}	13.3 ± 0.5^{cd}
	50%	11.3 ± 0.5^{df}	13.1 ± 0.1^{d}
Control MRS (no treatment)	None	12.3 ± 0.5^{de}	13.3 ± 0.5^{cd}

Data represent the mean ± standard error.
